# Multidimensional analysis of tumor stem cells: from biological properties, metabolic adaptations to immune escape mechanisms

**DOI:** 10.3389/fcell.2024.1441081

**Published:** 2024-08-08

**Authors:** Han Han, Ting He, Yingfan Wu, Tianmei He, Weiqiang Zhou

**Affiliations:** ^1^ Department of Biochemistry and Molecular Biology, Shenyang Medical College, Shenyang City, China; ^2^ Department of Pathogen Biology, Shenyang Medical College, Shenyang City, China

**Keywords:** tumor stem cells, biological properties, metabolic remodeling, immune escape, PD-L1

## Abstract

As a key factor in tumorigenesis, progression, recurrence and metastasis, the biological properties, metabolic adaptations and immune escape mechanisms of CSCs are the focus of current oncological research. CSCs possess self-renewal, multidirectional differentiation and tumorigenicity, and their mechanisms of action can be elucidated by the clonal evolution, hierarchical model and the dynamic CSCs model, of which the dynamic model is widely recognized due to its better explanation of the function and origin of CSCs. The origin hypothesis of CSCs involves cell-cell fusion, horizontal gene transfer, genomic instability and microenvironmental regulation, which together shape the diversity of CSCs. In terms of classification, CSCs include primary CSCs (pri-CSCs), precancerous stem cells (pre-CSCs), migratory CSCs (mig-CSCs), and chemo-radiotherapy-resistant CSCs (cr-CSCs and rr-CSCs), with each type playing a specific role in tumor progression. Surface markers of CSCs, such as CD24, CD34, CD44, CD90, CD133, CD166, EpCAM, and LGR5, offer the possibility of identifying, isolating, and targeting CSCs, but the instability and heterogeneity of their expression increase the difficulty of treatment. CSCs have adapted to their survival needs through metabolic reprogramming, showing the ability to flexibly switch between glycolysis and oxidative phosphorylation (OXPHOS), as well as adjustments to amino acid and lipid metabolism. The Warburg effect typifies their metabolic profiles, and altered glutamine and fatty acid metabolism further contributes to the rapid proliferation and survival of CSCs. CSCs are able to maintain their stemness by regulating the metabolic networks to maintain their stemness characteristics, enhance antioxidant defences, and adapt to therapeutic stress. Immune escape is another strategy for CSCs to maintain their survival, and CSCs can effectively evade immune surveillance through mechanisms such as up-regulating PD-L1 expression and promoting the formation of an immunosuppressive microenvironment. Together, these properties reveal the multidimensional complexity of CSCs, underscoring the importance of a deeper understanding of the biology of CSCs for the development of more effective tumor therapeutic strategies. In the future, therapies targeting CSCs will focus on precise identification of surface markers, intervention of metabolic pathways, and overcoming immune escape, with the aim of improving the relevance and efficacy of cancer treatments, and ultimately improving patient prognosis.

## 1 Introduction

Tumors are abnormal tissues that exhibit unrestricted growth and resilience to various survival stresses. In recent years, advancements in biotechnology and tumor biology have led to significant progress in the clinical treatment of tumors. However, the recurrence and metastasis of malignant tumors remain major obstacles to effective treatment. The high mortality rate among patients with malignant tumors presents a significant challenge. Tumor stem cells have emerged as a key focus of recent tumor theory research, being viewed as the primary ‘driving force’ behind tumor development and progression within tumor tissues, as well as the fundamental cause of tumor recurrence and metastasis (Islam et al., 2015). With their robust self-renewal and differentiation capabilities, tumor stem cells (CSCs) give rise to a diverse population within the tumor, playing a central role in the malignant traits of tumors such as proliferation, invasion, drug resistance, metastasis, and recurrence. This challenges the conventional theory of tumors arising randomly.

In the context of the CSCs model, tumor tissues harbor a subset of cells that, akin to normal stem cells, possess the ability to self-renew and differentiate into various cell types, thereby playing a role in tumor development. The mechanism of CSCs has been elucidated through three models: the clonal evolution model, the hierarchical CSCs model, and the dynamic CSCs model. Among these, the dynamic CSCs model is now considered more plausible due to its comprehensive explanation of the functional characteristics and source of CSCs.

1. Clonal evolution model: this model proposes that tumors consist of cells with the same genetic and epigenetic characteristics, and the homogeneity of these cells contributes to tumor development.
2. Hierarchical CSCs model: cancer stem cells (CSCs) play a crucial role in the initiation, maintenance, and progression of tumors. Less than 5% of CSCs at the top layer are responsible for tumor growth, capable of symmetrically dividing to produce identical daughter stem cells or undergoing asymmetric division to generate progenitor and stem cells.
3. Dynamic CSCs model: the dynamic CSCs model highlights the adaptability and diversity of the CSC population within the tumor microenvironment (Vermeulen et al., 2012). Experimental evidence shows that tumor cells can toggle between stem cell-like and differentiated states due to cytokines from mesenchymal stromal cells, indicating a reversible transition pathway between CSCs and non-CSCs. This implies that CSCs can differentiate into non-stem cell-like tumor cells and *vice versa*, showcasing a constantly evolving, genetically and phenotypically varied CSC population that mirrors the intricate and dynamic nature of CSCs.

## 2 Origin and classification of tumor stem cells

In addition to self-renewal, multidirectional differentiation, and tumorigenicity, some CSCs have the ability to migrate and are resistant to radiotherapy and chemotherapy. As a result, CSCs may develop into various types with distinct properties during self-renewal. While the CSCs theory has garnered support from numerous experiments, there are still differing perspectives on the origin of CSCs and the mechanisms of tumorigenesis. The stem cell origin theory posits that CSCs originate from normal stem cells following a series of genetic and epigenetic changes (Reya et al., 2001), whereas the cell fusion origin theory suggests that CSCs result from cell fusion (Shabo et al., 2020). Both of these viewpoints are backed by some experimental evidence.

### 2.1 Tumor stem cell origin hypothesis

The cancer stem cell (CSC) concept has been extensively studied in tumor biology, with several hypotheses proposed to explain their origin. One hypothesis, known as the cell-cell fusion hypothesis, suggests that CSCs may arise from the fusion of normal cells with tumor cells or stem cells with differentiated cells, leading to the introduction of new genetic combinations. Another hypothesis, the horizontal gene transfer hypothesis, focuses on the non-reproductive transfer of foreign gene fragments between cells, potentially conferring non-stem cell characteristics with stem cell-like properties. The genetic instability hypothesis proposes that the accumulation of genetic variations can disrupt normal cellular regulation, leading to the transformation of cells into CSCs with self-renewal abilities. Additionally, the microenvironmental hypothesis highlights the role of local environmental factors, such as inflammation, hypoxia, and cell-signaling molecules, in inducing non-CSCs to acquire or maintain stem cell properties. These diverse mechanisms collectively contribute to the complex and multifaceted process of CSC formation.

#### 2.1.1 Cell-cell fusion

Cell-cell fusion is a significant concept in investigating the origin of Cancer Stem Cells (CSCs). This phenomenon involves the merging of normal stem cells with transformed cells, resulting in the creation of new cells possessing both self-renewal capabilities and malignant transformation potential. This process not only plays a crucial role in tumor evolution but also significantly impacts various biological functions such as tissue formation, immune regulation, and tissue regeneration. In the maintenance of CSCs, the stem cell component ensures self-renewal capacity, while the genetic and epigenetic characteristics of differentiated cells contribute to specific histological features (Bjerkvig et al., 2005). Furthermore, the acquisition of stem cell properties by differentiated cells, either through oncogenic mutations or fusion with other differentiated cells carrying different oncogenic mutations, can provide alternative pathways for CSC formation. It is important to note that for this mechanism to be effective, at least one of the cells involved must harbor a key oncogenic mutation; otherwise, cell fusion may impede rather than promote tumorigenesis.

**FIGURE 1:**
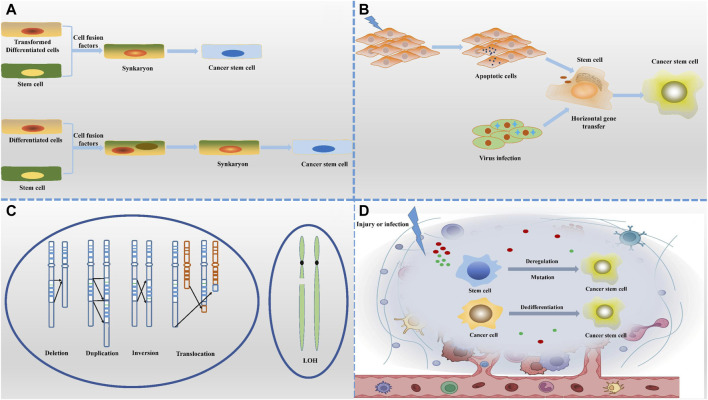
Origin of cancer stem cells. **(A)** The cell-cell fusion hypothesis suggests that CSCs may originate from the fusion of normal cells with tumour cells or stem cells with differentiated cells, a process that introduces new genetic combinations. **(B)** The horizontal gene transfer hypothesis highlights the non-reproductive transfer of foreign gene fragments between cells, which may confer stem cell-like characteristics to non-stem cell properties. **(C)** The genetic instability hypothesis states that the accumulation of genetic variants leads to dysregulation of normal cells that transform into CSCs with self-renewal capacity. **(D)** The microenvironmental hypothesis emphasizes that local environmental factors, such as inflammation, hypoxia and cell signaling molecules, can induce non-CSCs to acquire or maintain stem cell characteristics. Together, these diverse mechanisms of origin depict a complex and varied context for the formation of CSCs.

The hypothesis presented in Figure 1 suggests that cancer stem cells (CSCs) may arise from the fusion of normal stem cells with transformed cells, facilitated by cell fusion factors (Ogle et al., 2005). This fusion process gives rise to new cell types containing mono- or multinucleated structures. Notably, syncytiotrophoblasts form due to chromosome loss, with heteronucleosomes playing a role as a transitional state. It is believed that chromosome reduction initiates the development of CSC characteristics. The increasing occurrence of cell fusion is closely linked to the mechanism of cancer progression, indicating its significance in tumor evolution. Many cells found within tumors are hybrids of normal and tumor cells, exhibiting heightened malignant traits that contribute to tumor heterogeneity and aggressiveness. This hypothesis not only elucidates the mechanisms driving cancer diversity but also offers fresh perspectives on investigating the origins and advancement of cancer.

The cell fusion theory offers a logical explanation for the early occurrence of aneuploidy and the progression of tumors toward malignancy (Heselmeyer et al., 1996). It suggests that the aneuploid state resulting from chromosome rearrangement leads cells to develop malignant traits like uncontrolled growth and resistance to cell death, while also fostering genetic diversity within tumors. The theory further explains why certain primary tumors are more likely to spread to other parts of the body, particularly when fusion events involve cells capable of migration, such as macrophages (Chen and Olson, 2005). In these cases, the resulting cancer stem cells (CSCs) quickly exhibit a high potential for metastasis, especially to specific organs, reflecting the intrinsic characteristics of the fused cells.

The mechanism of cell fusion is linked to the selectivity of organs for tumor metastasis, with different types of tumors showing a preference for specific distant organs (Peng et al., 2021). This indicates that the characteristics of fused cells and the molecular markers they carry may determine the organ targeted for metastasis.

In normal physiological conditions, specific molecules are involved in the process of cell fusion. CD44, a marker for many cancer stem cells, is also thought to play a role in the creation of cancer stem cells through cell fusion (Hassn Mesrati et al., 2021). Furthermore, interleukin IL-4, crucial for muscle cell fusion, suggests that similar mechanisms may regulate the fusion of tumor cells with other cell types (Horsley et al., 2003). These discoveries not only enhance our understanding of the intricate mechanisms of tumor metastasis but also lay the groundwork for developing innovative therapeutic approaches that target cell fusion.

#### 2.1.2 Horizontal gene transfer

Horizontal gene transfer (HGT) is a unique genetic process that plays a significant role in the emergence of cancer stem cells (CSCs) (Bergsmedh et al., 2001; Bergsmedh et al., 2002). Unlike traditional vertical inheritance, HGT allows genes to be directly exchanged between unrelated individuals. While this phenomenon is more commonly observed in prokaryotes, its impact on eukaryotes, particularly multicellular organisms, is becoming increasingly recognized.

In the context of CSC formation, the horizontal oncogene transfer hypothesis suggests that mutations in somatic cells can lead to apoptosis, releasing DNA fragments containing mutated genes. These fragments may be taken up by neighboring normal stem or progenitor cells through processes like phagocytosis or endocytosis. Once integrated into the stem cell genome, these foreign DNAs can induce genetic reprogramming, activate or suppress key genes, and confer cancer stem cell traits such as self-renewal and enhanced migration capabilities.

It has been shown that apoptotic bodies from tumour cells exhibit a potential role in tumour development, as they not only induce colony formation in p53-deficient fibroblasts *in vitro*, but also promote tumour formation *in vivo*. This phenomenon reveals the importance of horizontal gene transfer in tumourigenesis and progression, whereby intact chromosomes or chromosome fragments are able to be transferred from apoptotic tumour cells to recipient cells via the phagocytic pathway (Bergsmedh et al., 2001; Bergsmedh et al., 2002). This non-traditional way of exchanging genetic material may provide new genetic information to tumour cells, which in turn affects their growth characteristics and promotes tumour progression.

It is worth mentioning that apoptotic bodies belong to a class of extracellular vesicles, and extracellular vesicles (EVs), as a carrier of intercellular communication, are rich in proteins, lipids, and nucleic acids, which are capable of being captured by other cells and thus affecting the function of recipient cells. MSCs are favoured donor cells for EVs due to their remarkable ability to generate EVs. In the study, bone marrow-derived MSC EVs were used as carriers and successfully transported miR-124a to glioma stem cells (GSCs). In this process, miR-124a specifically reduced the expression level of FOXA2, which in turn triggered the abnormal accumulation of lipids inside GSCs and affected their lipid metabolic homeostasis. This mechanism effectively inhibited the activity of GSCs and significantly prolonged the survival time of experimental mice (Lang et al., 2018). In addition, the study conducted by Ding et al. focused on human umbilical cord MSC-derived EVs, which were used to carry miR-145-5p and effectively reduce Smad3 expression. This manipulation interfered with the TGF-β-mediated epithelial mesenchymal transition process, which enhanced apoptosis and caused cell cycle arrest in pancreatic ductal adenocarcinoma cells, ultimately inhibiting tumour cell proliferation and invasion (Ding et al., 2019).

In addition, EVs have been shown to transfer microRNA-222 from mesenchymal stem cells to colorectal cancer cells, leading to immune escape (Li et al., 2021). In the tumour microenvironment, EVs secreted by cancer cells and immune cells play a key role in immune regulation. Cancer cell-derived EVs, which usually express programmed death ligand 1 (PD-L1) on their surface, are able to bind to PD-1 receptors on T cells and inhibit T cell activation and proliferation, thus weakening the immune system’s ability to recognise and clear tumour cells. This mechanism allows tumour cells to escape immune surveillance and promotes tumour growth and proliferation (Haderk et al., 2017; Chen et al., 2018; Gabrusiewicz et al., 2018; Morrissey and Yan, 2020). The extracellular vesicles released by CSC, when taken up by myeloid cells, promote the production of IL-6, IL-10 and IL-1β, thus further inhibiting T cell function (Wei et al., 2010; Volonté et al., 2014; Yao et al., 2016; Wu et al., 2017; Gabrusiewicz et al., 2018).

In addition, immune cell-derived EVs are involved in tumour immunomodulation. They may carry molecules capable of activating or inhibiting the immune response, affecting the status of surrounding immune cells, including enhancing or inhibiting the function of T cells, natural killer cells (NK cells) and other immune cells (Cai et al., 2021). By transmitting specific signals, these EVs modulate the activity of immune cells, which in turn affect the tumour immune environment and may promote or inhibit tumour progression.

In addition, viral infections can also propagate oncogenes to stem cells through the process of lysis, promoting their malignant transformation, and revealing the catalytic role of viruses in promoting the horizontal transfer of genetic material in some cancers (Holmgren et al., 1999). Thus, horizontal gene transfer is not only a key component of microbial evolution, but also plays an important role in the development of tumors in higher organisms, especially by altering stem cell properties. This mechanism reveals the deeper causes of tumor diversity and treatment resistance, and opens up new directions for cancer research and therapeutic strategies.

#### 2.1.3 Genetic instability

Manifested through abnormal chromosome genomic instability and genetic variation are fundamental aspects of cancer development, numbers (aneuploidy) and point mutations that impact the function of key genes (Sen, 2000; Gordon et al., 2012). Aneuploidy can lead to genomic imbalance, particularly the loss of oncogenes, which enhances cellular vulnerability to oncogenic factors and accelerates tumor progression. While most harmful mutations are typically eliminated by cellular processes, some mutations may accumulate, especially in the presence of carcinogens or when cellular protective mechanisms are compromised. Furthermore, epigenetic modifications can drive abnormal cell proliferation, creating a conducive environment for the accumulation of mutations. While chromosomal abnormalities and point mutations play crucial roles in the initial stages of tumor formation, the rarity of normal cellular carcinogenesis suggests the existence of additional unidentified mechanisms influencing the initiation and progression of tumors.

#### 2.1.4 Microenvironment

The presence of genetic mutations and chromosomal abnormalities in stem, progenitor, and differentiated cells can potentially lead to the formation of cancer stem cells (CSCs), a process that is heavily influenced by the cellular microenvironment. This microenvironment not only controls the differentiation of stem cells but also creates conditions that trigger the generation of CSCs by releasing signals that prompt stem cells to proliferate or dedifferentiate in response to specific stimuli, such as tissue damage or infection (Tu et al., 2023).

Under specific microenvironmental conditions, differentiated cells have the capability to revert to cancer stem cells (CSCs). The presence of an inflammatory microenvironment plays a significant role in promoting tumor initiation through the release of inflammatory cytokines, chemokines, and DNA damage factors. Inflammatory cytokines like IL-6 not only contribute to the generation of CSCs, but also play a role in regulating the equilibrium between CSCs and non-stem cell cancer cells (Tsai et al., 2011). Normally, CSCs can transition into non-stem cell cancer cells and *vice versa*, albeit with lower efficiency. However, factors like IL-6 produced by CSCs can facilitate the dedifferentiation of non-stem cell cancer cells. Research has shown that molecules such as IL-6 and nuclear factor κB (NF-κB) are instrumental in maintaining a proportional balance between CSCs and non-stem cell cancer cells in specific cellular contexts (Iliopoulos et al., 2009).

Immune cells such as macrophages and lymphocytes, growth factors, proteases, and cell-free signaling molecules present in the microenvironment play crucial roles in regulating stem cell differentiation. However, they can also lead to cell fusion, DNA mutations, and chromosomal abnormalities, ultimately contributing to the generation of cancer stem cells (CSCs) and tumorigenesis. Therefore, a comprehensive understanding of the regulatory mechanisms within the tumorigenic microenvironment is vital for the advancement of more efficacious tumor intervention strategies.

### 2.2 Classification of tumor stem cells

Some scholars categorize cancer stem cells (CSCs) into various types, such as primary cancer stem cells (primary-CSCs), precancerous stem cells (pre-CSCs), migrating cancer stem cells (mig-CSCs), and chemo- and radio-resistant cancer stem cells (cr-CSCs and rr-CSCs).

#### 2.2.1 pri-CSCs

In 1997, Bonnet and Dick utilized flow cytometry to identify a subpopulation of leukemia cells characterized by stem cell-like features, specifically CD34^+^CD38-cells. This discovery indicated the presence of cells within malignant tumors capable of self-renewal and multidirectional differentiation, believed to be fundamental to tumorigenesis (Bonnet and Dick, 1997). These findings supported the concept of Cancer Stem Cells (CSCs), a subset of tumor cells possessing stem cell properties that drive tumor growth and contribute to recurrence. Subsequent studies have sought similar CSCs in various solid tumors, such as CD44^+^CD24^−^ cells in breast cancer, referred to as ‘primary CSCs’ (pri-CSCs). These cells play a crucial role in tumor initiation and maintenance, often expressing distinct surface markers that facilitate their identification and isolation from heterogeneous populations of tumor cells.

#### 2.2.2 pre-CSCs

Pre-Cancer Precursor Stem Cells (pre-CSCs) have been identified in precancerous lesions and are considered as precursors to Primary Cancer Stem Cells (pri-CSCs). These pre-CSCs exhibit typical stem cell characteristics by expressing key stem cell maintenance factors like OCT3/4, SOX2, and KLF4, enabling them to self-renew and differentiate in multiple directions (Chen et al., 2007). Despite possessing stem cell properties, pre-CSCs do not directly give rise to tumors in immunocompetent hosts. Their developmental fate is tightly regulated by the microenvironment, allowing them to transform into either benign or malignant cells.

As the disease progresses to the cancer stage, pre-CSCs undergo a critical transition to evolve into pri-CSCs, which play a key role in promoting tumor formation. Pri-CSCs are specifically found in formed tumor tissues, losing their ability to transform bidirectionally and instead driving the development of malignant tumors exclusively compared to pre-CSCs. This transition underscores the different roles and functional differences between pre-CSCs and pri-CSCs in the continuum of tumor development, with pre-CSCs representing a transitional state from normal to abnormal and pri-CSCs being a central driver of malignant tumor development.

These findings underscore the significance of detecting and addressing pre-cancer stem cells (pre-CSCs) at various disease stages, particularly in the initial phases of cancer. The adaptable nature of this population could offer a valuable opportunity for therapeutic intervention to either inhibit cancer development or impede its advancement. Furthermore, a comprehensive comprehension of the molecular mechanisms involved in the transition from pre-CSCs to pri-CSCs, as well as the influence of the microenvironment on this transition, is crucial for devising targeted therapeutic approaches against cancer stem cells.

#### 2.2.3 mig-CSCs

Metastasis is a crucial stage in the spread of malignant tumors from the original site to other parts of the body. The ‘seed-soil’ theory illustrates the specific affinity between a tumor cell (seed) and the microenvironment of a distant organ (soil) (Fidler, 2003). Cancer stem cells (CSCs) are considered as potential ‘seeds’ in metastasis due to their ability to self-renew, proliferate continuously, and adapt well to external stresses. Not all CSCs have the same metastatic potential; certain subpopulations, like those expressing markers such as CD133 and CXCR4, are identified as migratory cancer stem cells with high metastatic capacity (mig-CSCs) (Hermann et al., 2007). These mig-CSCs are crucial in driving metastasis in various types of cancer.

The lack of highly specific biomarkers makes it challenging for researchers to accurately identify mig-CSCs, hindering their exploration of these subpopulations of cells with metastatic potential. Epithelial mesenchymal transition (EMT) is a crucial biological process that enables epithelial cells, initially attached to the tissue substrate, to acquire migratory and invasive capabilities. This transformation may facilitate the conversion of CSCs into metastasis-competent mig-CSCs, offering valuable insights into the process of how CSCs develop into a subpopulation of cells with metastatic potential.

In-depth exploration of the specific properties of mig-CSCs, identification of their unique biomarkers, and unraveling the molecular mechanisms that regulate their metastatic ability are crucial for understanding the complex process of cancer metastasis. This research will not only enhance our understanding of the molecular basis of metastasis but also potentially lead to the development of new strategies to inhibit metastasis and personalized therapeutic approaches targeting mig-CSCs. Ultimately, this could significantly improve the prognosis and quality of life for cancer patients.

#### 2.2.4 cr-CSCs and rr-CSCs

The identification of drug-resistant cancer stem cells (cr-CSCs) originated from research on colon cancer cells resistant to chemotherapy, which maintain their resistance through autocrine interleukin-4 (IL-4) signaling to increase anti-apoptotic protein expression (Todaro et al., 2007). These cells can be distinguished by the CD133+CD24low marker. While numerous studies have investigated the interplay between cancer stem cells and chemotherapy, the precise role of cr-CSCs and their prevalence require further comprehensive validation. Such confirmation is essential to establish a robust theoretical foundation for the advancement of new tumor treatment strategies and targeted therapies.

Breast cancer stem cells exhibit lower levels of reactive oxygen species (ROS) compared to differentiated cells, along with a more efficient antioxidant system that helps mitigate radiation-induced DNA damage. This difference in ROS levels may contribute to the resistance of cancer stem cells (CSCs) to chemotherapy and their potential tolerance to radiation (Diehn et al., 2009). Glioma and breast cancer stem cells increase autophagic protein expression and activate DNA repair mechanisms post-radiation to evade the cytotoxic effects of chemotherapy. Conversely, elevated ROS-induced DNA damage aids in the elimination of CSCs. The presence of diverse subpopulations of primitive cancer stem cells (pri-CSCs), including chemo-resistant and radiation-tolerant subtypes, remains an open question that necessitates further investigation. Current research aims to address this query, emphasizing the need for more comprehensive scientific studies to elucidate this hypothesis.

## 3 Tumor stem cell surface markers

Cancer stem cells (CSCs) exhibit specific biomolecules highly expressed on their cell membrane, often linked to maintaining stemness and tumorigenic properties. These markers serve purposes such as isolation, identification, and targeted therapy of CSCs. As research on CSCs progresses, more potential surface markers have been identified, including CD24, CD34, CD44, CD90, CD133, CD166, EpCAM, and LGR5. These markers are crucial in various tumor types (Sipos and Mûzes, 2023).

### 3.1 CD24

CD24, a small glycophosphatidylinositol (GPI)-anchored protein, was initially studied in immunology for its presence on the surface of Ig-positive B cells and mature granulocytes (Ni et al., 2020), shedding light on the intricate functions of cell surface molecules in the immune system (Panagiotou et al., 2022). More recently, CD24 has garnered attention in oncology, particularly for its role in identifying cancer stem cells (CSCs). The expression of CD24 in various malignant tumors, such as breast, gastric, hepatocellular, and bladder cancers, is closely linked to tumor invasiveness, metastatic potential, and response to treatment, suggesting a pivotal role in tumor progression (Choi et al., 2009). Consequently, CD24 not only enhances our comprehension of tumor biology mechanisms, but also emerges as a potential tumor marker and a novel target for therapeutic interventions. This has significant implications for advancing early cancer diagnosis and tailoring personalized treatment approaches.

### 3.2 CD34

CD34 is a type I transmembrane glycoprotein that is highly glycosylated and associated with endothelial cells and angiogenesis. It was the first surface marker identified for Cancer Stem Cells (CSCs), originating from a 1994 study by Lapidot et al. (1994). In this study, a population of CD34^+^ CD38-cells was isolated from acute myeloid leukemia samples (Uckun et al., 1995). These cells were found to have the ability to trigger acute leukemia after transplantation into immunodeficient mice, displaying characteristics of self-renewal and high tumor-forming capacity. This led to the establishment of the concept of leukemic stem cells (LSCs).

### 3.3 CD44

CD44, a transmembrane glycoprotein encoded by a gene on human chromosome 11, forms multiple heterodimers with varying functions due to different splicing patterns (Zheng et al., 2022). It is involved in crucial cellular activities such as cell interaction with extracellular matrix components like hyaluronic acid and regulation of cell adhesion, migration, proliferation, and differentiation (Wielenga et al., 1999; Hao et al., 2010; Ishimoto et al., 2011). CD44 is essential for normal stem cell maintenance and homing, and is a distinguishing feature of cancer stem cells (CSCs) in various cancers including lung, melanoma, leukemia, and breast cancers (Du et al., 2008; Bapat, 2010). In malignant tumors, abnormal CD44 expression is linked to aggressive growth and metastasis. By interacting with signaling molecules like c-Src, CD44 influences cell signaling pathways, promoting self-renewal and differentiation of tumor stem cells, thereby contributing significantly to tumorigenesis and progression.

Furthermore, CD44 is associated with the dysregulation of the Wnt/β-catenin signaling pathway, which is more active in CD44 and CD133 enriched cells, further enhancing tumor proliferation and malignant transformation (Wielenga et al., 1999; Hao et al., 2010; Ishimoto et al., 2011). Given its critical role in tumor stem cell maintenance and tumor progression, CD44 has become an attractive target for cancer therapy. By regulating the expression level of CD44 or interfering with its downstream signaling pathways, scientists expect to develop effective strategies to inhibit tumor growth, which not only emphasizes the potential of CD44 as a therapeutic target, but also highlights its importance as a biomarker for predicting tumor prognosis. Therefore, an in-depth study of the function of CD44 and its mechanism of action in different tumor types is of great significance in promoting the development of precision medicine and cancer treatment strategies.

### 3.4 CD90

CD90, also known as Thy-1, is a cell surface molecule that is anchored to the cell membrane through glycosylphosphatidylinositol (GPI). Initially recognized as a key marker for identifying and distinguishing hematopoietic stem cells, further research has revealed that CD90 plays a significant role beyond the hematopoietic system. It has been shown to have important biological implications in various solid tumors. Specifically, CD90 has been identified as a distinguishing characteristic of cancer stem cells (CSCs) in malignancies such as hepatocellular carcinoma (Yang et al., 2008), gastric carcinoma, and certain types of leukemia. This discovery has broadened our understanding of the mechanisms underlying tumor initiation, progression, and resistance to treatment.

### 3.5 CD133

CD133, also known as Prominin1, is a glycoprotein encoded by a single-copy gene located on chromosome 4. It consists of 865 amino acids and 37 exons (Mia-Jan et al., 2013), with a complex structure that includes extracellular and intracellular regions, as well as transmembrane structures (Cui et al., 2010). Originally identified in neuroepithelial cells in 1997, CD133 is now considered a key marker of cancer stem cells (CSCs) in various malignant tumors like lung, colorectal, prostate, and liver cancers. Its expression is inversely correlated with cell differentiation and plays a crucial role in maintaining stemness and promoting tumor development.

In addition to its role in stem cells under normal conditions, CD133 has emerged as a valuable prognostic indicator and is widely used in cancer classification and diagnosis. While more research is needed to fully understand its functions and prognostic significance in different cancer types, CD133 has been recognized as a marker of CSCs in brain tumors, prostate cancers, and colon cancers, impacting patient survival outcomes (Mia-Jan et al., 2013; Zhang et al., 2016). Notably, in colon cancer, both CD133-positive and CD133-negative cells have shown tumorigenic potential, highlighting the multifaceted role of CD133 in cancer development (Mersakova et al., 2022).

### 3.6 CD166

CD166, also known as Activated Leukocyte Cell Adhesion Molecule (ALCAM), is a membrane protein that plays a crucial role in cell adhesion and migration (Yang et al., 2021). It is involved in regulating immune responses and facilitating intercellular communication through interactions with CD6 molecules on T lymphocytes. Recent studies have shown a strong correlation between the expression of CD166 and the presence of Cancer Stem Cells (CSCs) in various types of cancer, such as lung (El-Ashmawy et al., 2021), colorectal (Han et al., 2017), and gastric cancers (Nguyen et al., 2017), highlighting CD166 as a significant marker for CSCs in these malignancies.

### 3.7 EpCAM

EpCAM, also known as epithelial cell adhesion molecule, plays a crucial role in maintaining the structural integrity and function of epithelial tissues. It is involved in various physiological activities such as intercellular adhesion, proliferation, differentiation, and migration (Keller et al., 2019). Abnormal expression of EpCAM is a common feature in many cancers, particularly gastrointestinal tumors, suggesting its significance in tumorigenesis and cancer development.

EpCAM serves not only as a regulator of normal epithelial cell functions but also as a focal point in cancer research, being identified as a marker for cancer stem cells (CSCs) in solid tumors. For instance, in non-small cell lung cancer, EpCAM expression helps identify minor cell populations with self-renewal and tumor-forming capabilities, which are linked to tumor recurrence and metastasis (Masciale et al., 2020).

In colorectal cancer, EpCAM expression levels are associated with tumor aggressiveness, disease progression, and poor prognosis. Similarly, in liver cancer studies, EpCAM expression correlates with cancer progression and patient survival, highlighting its potential for diagnosing and assessing liver cancer prognosis (Krause et al., 2020). Moreover, in pancreatic cancer, EpCAM may facilitate tumor cell proliferation and migration, suggesting its involvement in the growth and metastasis of pancreatic tumors (Ishiwata et al., 2018).

### 3.8 LGR5

LGR5, the leucine-rich repeat sequence of G protein-coupled receptor 5, is a crucial molecule involved in regulating various biological processes, particularly in maintaining the stability of intestinal epithelial cells and controlling stem cell properties. Initially identified as a marker for gastrointestinal stem cells, LGR5 plays a direct role in the Wnt signaling pathway in colorectal cancer and small intestinal crypt cells (Barker et al., 2007), indicating its significance in stem cell self-renewal and suggesting its potential as a marker for CSCs.

Structurally, LGR5 is characterized by its N-terminal extracellular domain with multiple leucine repeats, enabling it to tightly bind growth factors in the R-spondin family (RSPO1 to RSPO4) (de Lau et al., 2011). The interaction of R-spondins with LGR5 and the LRP5/6 receptor significantly enhances the Wnt signaling pathway, leading to β-catenin accumulation, a critical step in regulating cell proliferation and stem cell self-renewal.

Table 1 outlines various CSC markers, showcasing the diverse and evolving range of markers in this field, reflecting the complexity and heterogeneity of tumors. The molecular diversity among CSC subpopulations necessitates more precise clinical strategies to identify and target these specific cell populations effectively. Integration of multiple markers allows scientists and clinicians to better profile CSCs and develop personalized therapeutic approaches, such as utilizing antibody-drug conjugates (ADCs) to target CSCs with specific surface antigens or creating innovative immunotherapies to trigger immune responses against CSCs.

**TABLE 1:** Cell surface markers expresses on cancer stem cells.

Maker	Type	Cancers
CD24	Membrane proten	Breast Cancer, Stomach Cancer, Liver Cancer, Bladder Cancer, Lung Cancer, Pancreatic Cancer, Colorectal Cancer, Ovarian Cancer, Head and Neck Cancer, Stomach Cancer, Oesophageal Cancer
CD34	Membrane proten	Liver Cancer, Non-Small Cell Lung Cancer
CD44	Membrane proten	Breast Cancer, Lung Cancer, Colorectal Cancer, Prostate Cancer, Liver Cancer, Pancreatic Cancer, Ovarian Cancer, Blood Cancer, Brain Cancer, Head and Neck Cancer, Bladder Cancer, Stomach Cancer, Cervical Cancer, Oesophageal Cancer
CD90	Membrane proten	Liver Cancer, Stomach Cancer, Leukaemia, Breast Cancer, Liver Cancer, Lung Cancer, Blood Cancer, Brain Cancer, Head and Neck Cancer, Stomach Cancer, Oesophageal Cancer
CD133	Membrane proten	Lung Cancer, Colorectal Cancer, Prostate Cancer, Liver Cancer. Pancreatic Cancer, Renal Cell Cancer, Brain Cancer, Liver Cancer, Lung Cancer, Colon Cancer, Pancreatic Cancer, Prostate cancer, Ovarian cancer, melanoma, Head and Neck cancer, Brain cancer, Stomach cancer, Kidney cancer
CD166	Adhesion molecule	Lung cancer, Colorectal cancer, Stomach cancer, Prostate cancer, Melanoma cancer
EpCAM	Adhesion molecule	Non-small cell lung cancer, Colorectal cancer, Liver cancer, Pancreatic cancer, Breast cancer, Liver cancer, Lung cancer, Pancreatic cancer, Colon cancer, Ovarian cancer, Brain cancer, Stomach cancer
LGR5	Receptor protein	Breast cancer, Stomach cancer, Colon cancer, Head and Neck cancer
CD19	Membrane proten	Blood cancer
CD20	Membrane proten	Blood cancer, melanoma
CD38	Membrane proten	Blood cancer
ABCB5	Transporter	Melanoma

While the therapeutic strategies discussed demonstrate promise, the identification and utilization of surface markers for CSCs present significant challenges. The variability in CSCs marker expression and the heterogeneity among cell populations hinder the use of a single marker as a universal identifier for CSCs. Moreover, the lack of universally applicable CSCs markers across all cancer types complicates treatment approaches. Thus, ongoing scientific investment and technological advancements are crucial to uncover more reliable CSCs markers, enhance the combined use of existing markers, and deepen our understanding of CSCs’ biological properties. These efforts aim to propel cancer treatment forward, enhancing precision and efficacy in therapeutic interventions to ultimately improve cancer patients’ survival rates and quality of life. Future cancer treatment strategies will focus on personalization and targeting, necessitating continual innovation and application grounded in a comprehensive comprehension of CSCs markers.

## 4 Metabolic profile of tumor stem cells

Research in the field of tumor metabolism has elucidated how tumor cells, including tumor stem cells, undergo metabolic reprogramming to fulfill their requirements for rapid proliferation and survival. This metabolic adaptation, known as metabolic reprogramming, is a fundamental process through which tumor cells adjust to their surroundings and enhance their own growth. The Warburg effect serves as a prominent example, illustrating tumor cells’ inclination towards glycolysis even in the presence of oxygen. This metabolic strategy not only supplies the energy necessary for rapid cell division but also furnishes essential building blocks for biosynthesis (Graziano et al., 2017). Furthermore, studies have revealed that alterations in tumor metabolism extend beyond glycolysis to encompass significant changes in lipid and amino acid metabolism, collectively contributing to tumor progression, drug resistance, and metastasis.

Notably, the metabolic characteristics of tumor stem cells play a crucial role in maintaining their stemness, resisting treatment, and fostering tumor recurrence and metastasis. Cancer stem cells adeptly adjust their metabolic networks to withstand adverse conditions and evade therapeutic pressures, regulating processes such as energy production, antioxidant defenses, epigenetic modifications, and signaling pathways. For instance, cancer stem cells may rely on glutamine metabolism to support nucleotide and amino acid synthesis for proliferation and DNA repair, or utilize fatty acid oxidation to generate energy and biosynthetic precursors, particularly in oxygen-deprived or nutrient-poor environments.

### 4.1 Energy metabolic properties of tumor stem cells, including glycolysis, oxidative phosphorylation (OXPHOS), and energy metabolic adaptations

Cancer stem cells (CSCs) demonstrate remarkable flexibility in their energy metabolism, crucial for their survival, self-renewal, and tumor progression. They exhibit a preference for glycolysis (known as the Warburg effect) in aerobic conditions, swiftly converting glucose to lactate to fuel rapid cell growth and biomolecule synthesis (Vlashi et al., 2011). Despite this, CSCs can also switch to oxidative phosphorylation (OXPHOS) when faced with environmental challenges or therapeutic pressures, bolstering their resilience and resistance to treatment. This dynamic ability to transition between glycolysis and OXPHOS, alongside the regulation of other metabolic pathways like amino acids and fatty acids, allows CSCs to efficiently generate energy and essential molecules in diverse microenvironments, sustain their stem cell characteristics, and drive tumor growth (Zhou et al., 2011; Liu et al., 2014; Hur et al., 2017; Peng et al., 2018).

#### 4.1.1 Glycolysis

**FIGURE 2:**
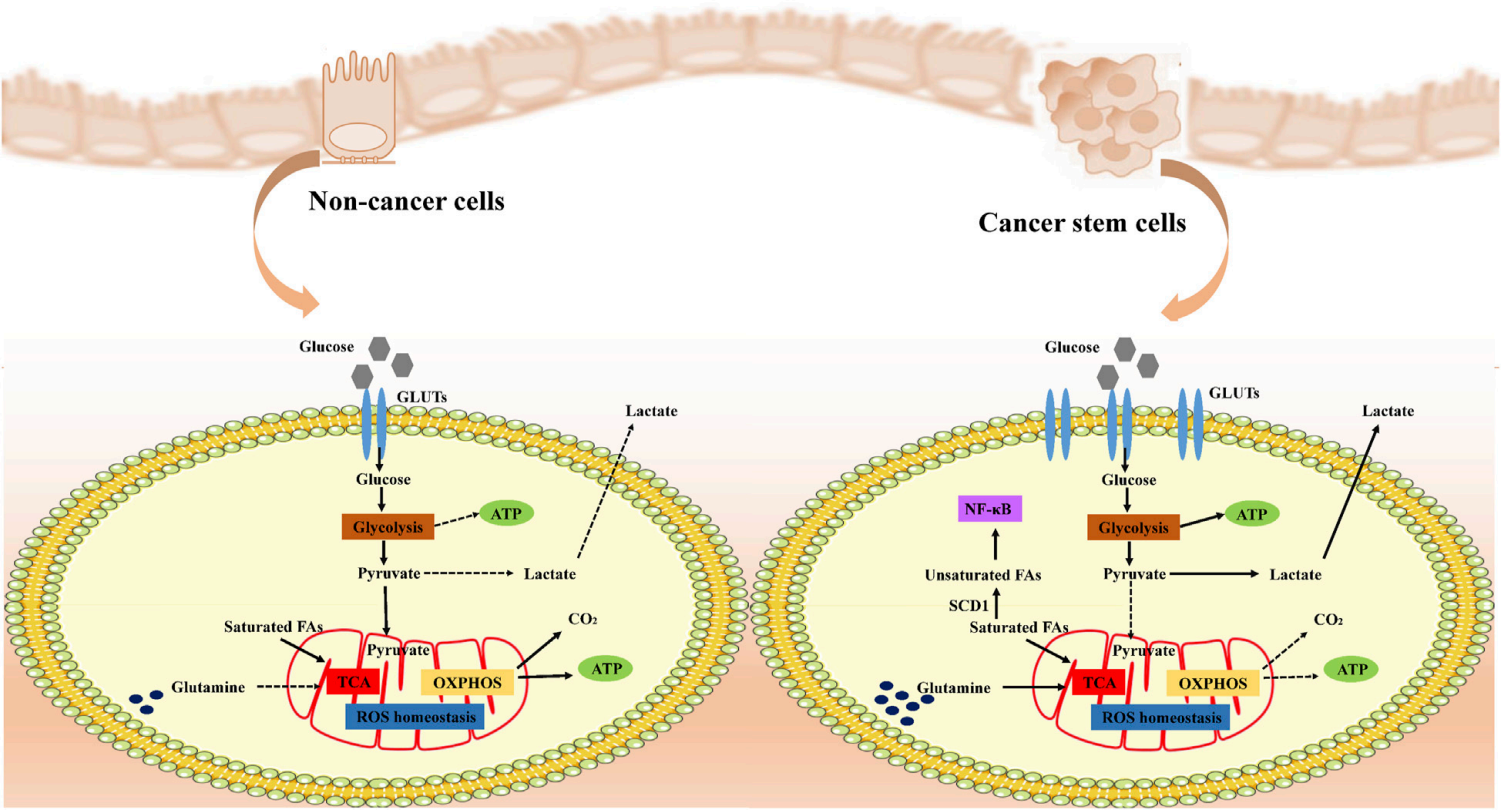
Metabolic remodeling of cancer cells. Cancer stem cells (CSCs) remodel their metabolic pathways to adapt to rapid proliferation and survival needs. Under normal conditions, cells efficiently produce ATP through aerobic respiration, whereas in cancer cells, even with sufficient oxygen, anaerobic glycolysis (Warburg effect) is preferred to produce lactic acid and maintain low levels of ROS, utilizing glycolysis-generated NADPH to strengthen antioxidant defenses and protect their stemness. CSCs specifically adapt amino acid metabolism to turn glutamine into a key metabolic pillar, both supplying energy and Supplementing TCA cycle intermediates, CSCs modulate the PI3K-Akt signaling pathway and influence cell proliferation and metabolism. In addition, CSCs alter fatty acid metabolism, especially increasing unsaturated fatty acid content, affecting cell membrane properties and signaling, which not only maintains their physiological activities, but may also be a novel strategy to intervene in the properties of CSCs and inhibit tumour progression. Overall, CSCs ensure efficient energy production and biosynthesis through these metabolic remodeling strategies to promote their survival and proliferation in resource-limited environments.

Cancer stem cells (CSCs) display distinct metabolic characteristics that support their rapid growth and preservation of stem cell properties (Figure 2). Initially, they boost glucose absorption by increasing the levels of transporter proteins like GLUT1 to fuel intense metabolic activity (Liu and Song, 2019). Subsequently, CSCs hasten the breakdown of glucose into pyruvate by overexpressing key glycolytic enzymes such as hexokinase and phosphofructokinase, not only producing ATP but also supplying ample reducing agents like NADH and NADPH to facilitate biosynthesis and maintain a reduced cellular environment. Despite adequate oxygen availability, CSCs favor glycolysis over oxidative phosphorylation, leading to the accumulation of lactate through the Warburg effect. This lactate not only helps regulate cellular pH but may also assist in evading immune surveillance. The excess lactate is then expelled from the cell through exocytosis. Moreover, CSCs keep reactive oxygen species (ROS) levels low, bolster antioxidant defenses with NADPH from glycolysis, and shield themselves from oxidative stress-induced harm, thereby safeguarding their stem cell characteristics and ensuring survival. In essence, these metabolic adaptations collectively contribute to the high energy production, resistance to oxidative stress, and rapid proliferation of CSCs, crucial for maintaining their properties and driving disease progression.

#### 4.1.2 Oxidative phosphorylation

Recent studies have shown that a subset of cancer stem cells (CSCs) may prefer oxidative phosphorylation (OXPHOS) over glycolysis for their energy production, exhibiting lower rates of glucose consumption and lactate production (Janiszewska et al., 2012; Lagadinou et al., 2013; Viale et al., 2014; Sancho et al., 2015). This metabolic state allows CSCs to generate more ATP efficiently, despite a slower rate of energy production per unit of glucose. This strategy supports long-term self-renewal, resistance to metabolic stress, and treatment resistance in CSCs.

Additionally, CSCs with a bias towards OXPHOS typically have higher mitochondrial mass and function, with mitochondria playing a key role in energy production, calcium storage, signal transduction, and apoptosis regulation. The enhanced mitochondrial activity not only reflects the energy efficiency of CSCs but also contributes to their ability to maintain stemness, promote survival, and adapt to challenging microenvironments.

#### 4.1.3 Energy metabolism adaptation

The metabolic profile of cancer stem cells (CSCs) is highly diverse and adaptive, with the ability to switch between glycolysis and oxidative phosphorylation (OXPHOS) based on their microenvironment. Variations in metabolic profiles can be observed even among different subpopulations of CSCs within the same tumor, such as mesenchymal-like and epithelial-like states in breast cancer stem cells. This metabolic flexibility extends not only between different CSC subpopulations but also among CSCs from different locations within the same tumor (Luo et al., 2018). For instance, glioma stem cells primarily use OXPHOS for energy production but can quickly switch to glycolysis when faced with metabolic stress like OXPHOS inhibition. This adaptive metabolic behavior is influenced by various regulatory mechanisms, including oncogenes, mitochondrial DNA mutations, genetic factors, and the cell cycle. Understanding these complex regulatory networks is essential for developing targeted therapeutic approaches for CSC-related tumors.

### 4.2 Characterization of substance metabolism, such as amino acid metabolism and lipid metabolism

Substance metabolism provides raw materials for the energy metabolism of CSCs and also plays a key role in maintaining the biological characteristics of CSCs.

#### 4.2.1 Amino acid metabolism

Amino acids serve as the fundamental building blocks for protein synthesis and are also involved in various non-protein metabolic processes such as energy production, redox state maintenance, and signaling. In cancer stem cells (CSCs), amino acid metabolism experiences significant changes, including the prioritization of essential amino acids like lysine and the transformation of the typically non-essential amino acid glutamine into a ‘quasi-essential’ amino acid crucial for supporting rapid cell proliferation. The unique metabolic adaptations seen in tumor cells, like the reliance on glutamine, allow them to thrive in challenging environments and sustain their high growth rates.

Glutamine not only provides nitrogen for cellular processes but also contributes carbon and reducing power to the tricarboxylic acid cycle, supporting biosynthesis and energy production. The breakdown of glutamine into glutamate and α-ketoglutarate plays a vital role in fueling the energy needs of rapidly dividing cancer cells by replenishing key intermediates in the TCA cycle. These metabolic adjustments enable tumor cells to maintain their active metabolism and growth despite unfavorable conditions. Moreover, glutamine indirectly impacts the PI3K-Akt signaling pathway by modulating mitochondrial ROS production, which in turn influences cell proliferation, survival, and overall metabolic balance (Deshmukh et al., 2016). Ultimately, tumor cells take advantage of the flexibility of glutamine metabolism to maintain high-intensity energy output and biosynthesis demand under resource-limited conditions, promoting their rapid growth and proliferation (Daye and Wellen, 2012).

#### 4.2.2 Lipid metabolism

The preservation of stemness in cancer stem cells (CSCs) is intricately linked to their distinct lipid metabolism profile. These cells utilize alterations in lipid metabolism to uphold their self-identity through various mechanisms, such as increasing fatty acid levels for energy provision, enhancing β-oxidation for energy efficiency, and activating the mevalonate pathway to boost cholesterol synthesis. When glycolysis is hindered in CSCs, lipid droplets (LDs) serve as an alternative energy source to shield fatty acids from oxidation. Additionally, the interplay between lipid metabolism and autophagy enables CSCs to swiftly adapt to the challenging environment post-chemotherapy.

Fatty acids (FAs) play a pivotal role in the biological functions of CSCs, serving as crucial components for constructing cell membranes, participating in signaling pathways, and supporting diverse cellular processes, particularly in energy metabolism. CSCs encounter rigorous microenvironmental stresses, relying heavily on mitochondria to efficiently metabolize nutrients like fatty acids into ATP, essential for sustaining cellular functions. This metabolic process unveils the distinctive adaptations of CSCs. Notably, the heightened levels of unsaturated fatty acids (UFA) in CSCs are closely associated with the actions of regulatory enzymes such as SCD1, ALDH1A1, and the inflammatory factor NF-κB (Sun and Yang, 2019). These alterations impact not only the fluidity and stability of cell membranes but also, through the modulation of specific signaling pathways, can significantly impede the stemness characteristics of CSCs, thereby suppressing tumor progression. This suggests that targeting UFA metabolism could be a potential avenue to intervene in CSC function. The capacity of CSCs to uphold fatty acid homeostasis is crucial for their metabolic robustness and resistance to anti-cancer treatments. Key fatty acid synthases (e.g., ACL, ACC, FASN) are active in CSCs and this process is regulated by the transcription factor SREBP1c, further highlighting the potential of these enzymes as therapeutic targets against the properties of CSCs (Rivas Serna et al., 2020).

Reduced activity of the energy receptor AMPK in CSCs leads to enhanced lipolysis, providing abundant precursors for fatty acid synthesis and promoting fatty acid β-oxidation in mitochondria (Bort et al., 2020). This metabolic adaptive mechanism further strengthens the energy production and self-sustainability of CSCs, showcasing their exquisite metabolic flexibility (Lin et al., 2017). Through glycolipid-metabolism interactions, pyruvate produced by glycolysis enters the fatty acid synthesis pathway via acetyl coenzyme A, promoting the self-renewal of CSCs and the maintenance of stemness characteristics.

Cholesterol metabolism is crucial for cellular structure and function, with exogenous cholesterol coming from dietary intake and endogenous cholesterol being produced through biosynthetic pathways, notably the mevalonate (MVA) pathway. This pathway not only synthesizes cholesterol but also produces steroid hormones and non-sterol isoprenoids, essential for regulating physiological processes like cell growth, division, and signaling. The geranylation of proteins in this pathway is crucial for maintaining the microenvironment of CSCs (Mullen et al., 2016). Overexpression of HMG-CoA reductase in certain basal-like tumors may be linked to tumor aggressiveness, drug resistance, and poor prognosis (Mancini et al., 2018). Regulating cholesterol metabolism by interfering with HMG-CoA reductase activity could significantly impact CSCs and their microenvironment by influencing cell signaling, cell cycle progression, and apoptosis pathways, opening up new possibilities for treatment.

## 5 Immune escape mechanisms of tumor stem cells

**FIGURE 3:**
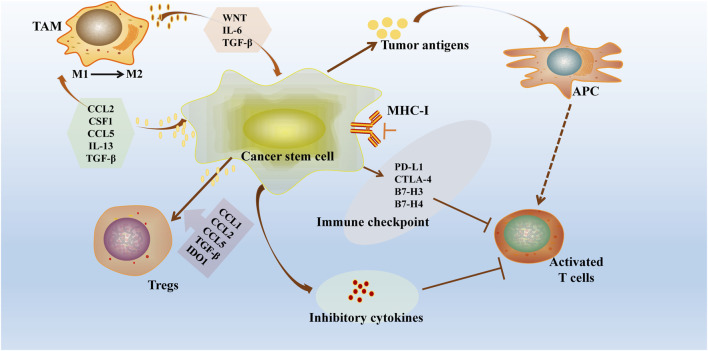
Immune escape mechanisms of tumour stem cells. CSCs exhibit significant immune resistance properties in different tumour types through several mechanisms: downregulation of antigen loss or antigen presentation mechanisms, construction of an immunosuppressive microenvironment (recruitment of immune cells and release of immunosuppressive cytokines), overexpression of inhibitory immune checkpoints and exploitation of auto-oncogenic signaling pathways. CSCs downregulate the MHC-I and overexpress checkpoint regulators, including PDL1. CSCs further drive Treg cell recruitment and polarisation through the binding of CCL1, CCL2, CCL5, TGF-β and indoleamine 2,3-dioxygenase 1 (IDO1). These strategies not only protect CSCs from recognition and clearance by T cells and NK cells, but also allow them to survive in a resting or dormant state, promoting tumour recurrence and metastasis.

Recent studies have highlighted the immune resistance properties of Cancer Stem Cells (CSCs) in various tumor types (Huang et al., 2020). These properties are primarily achieved through mechanisms such as downregulation of antigen expression, creation of an immunosuppressive microenvironment, upregulation of inhibitory immune checkpoints, secretion of immunosuppressive cytokines, and activation of self-oncogenic signaling pathways (Figure 3). These strategies not only shield CSCs from recognition and elimination by T cells and NK cells but also allow them to survive in a quiescent state, facilitating tumor recurrence and metastasis (Tsuchiya and Shiota, 2021). This emphasizes the critical need for therapeutic approaches targeting the immune evasion mechanisms of CSCs in cancer treatment.

### 5.1 CSCs downregulate antigen processing and presentation mechanisms

One of the primary strategies employed by cancer stem cells (CSCs) to evade detection by the immune system is to decrease their own antigenicity. This is achieved by inhibiting key components of the antigen processing and presentation pathways (Marzban et al., 2023). A common occurrence is the downregulation of antigen processing-associated transporter protein (TAP) and major histocompatibility complex class I (MHC-I). TAP plays a crucial role in transporting intracellular peptides to the endoplasmic reticulum for processing and binding to MHC-I molecules (Del Val et al., 2020). The resulting complexes are then presented on the cell surface for recognition by T cells. By reducing the expression of TAP and MHC-I, CSCs effectively diminish the presentation of self-antigens, thus evading immune surveillance by T cells.

Studies have shown. significant decrease in the expression of HLA-class I molecules, ALDH high CSCs/CICs may have the ability to evade immune surveillance, especially cytotoxic T-lymphocyte (CTL) attack (Shirosaki et al., 2024).

Increasing MHC-I expression could be a promising strategy to enhance the effectiveness of tumor immunotherapy (Gu et al., 2021). Despite the potential vulnerability of CSCs with reduced MHC-I levels to natural killer (NK) cells, some CSCs have developed additional immune evasion tactics). For instance, in conditions like acute myeloid leukemia, breast cancer, and glioblastoma, CSCs have been observed to downregulate the expression of NK cell-activating ligands (e.g., NKG2D ligands) and release ligands that inhibit NK cell recognition and killing, such as MICA, MICB, and ULBP1-6, enabling successful immune escape.

CSC are also capable of affecting NK cell activity. For example, CD133+ patient-derived glioblastoma stem cells are able to secrete TGFβ to inhibit NKG2D expression on peripheral blood monocytes, thereby affecting NK cell function (Beier et al., 2012).

In addition, CSC interfere with dendritic cell maturation through major histocompatibility complex class I antigen G (MHC-G)-immunoglobulin-like transcript (ILT) inhibitory receptor interactions (Grange et al., 2015).

### 5.2 CSC promotes the establishment of immunosuppressive tumor microenvironment

The tumor microenvironment and cancer stem cells (CSC) have intricate interactions with immune cells and their secreted factors. These interactions can lead to the creation of an immunosuppressive microenvironment and hinder the ability of cytotoxic immune cells to kill cancer cells, ultimately resulting in immune escape (Ruiu et al., 2019).

#### 5.2.1 Recruitment of immune cells

Polarization imbalance of tumor-associated macrophages (TAMs) in the tumor microenvironment, particularly the accumulation of M2-type TAMs, plays a significant role in cancer progression (Boutilier and Elsawa, 2021). These M2-type TAMs foster an immunosuppressive environment that supports tumor growth by releasing immunosuppressive factors like IL-10, CCL17, CCL22, as well as pro-angiogenic factors such as VEGF and PIGF. In specific scenarios like hepatocellular carcinoma, TAMs induce epithelial-mesenchymal transition of CSCs through TGF-β, while activating specific signaling pathways like EGF to enhance CSC proliferation, highlighting their crucial involvement in tumor invasion, metastasis, and acquisition of stem cell characteristics (Fan et al., 2014). Moreover, TAMs interact with various tumor cells and trigger multiple signaling pathways via intricate paracrine signals such as IL-6, MFGE8, and CCL5 to boost stem cell self-renewal and resistance to chemotherapy (Gao et al., 2022), with STAT3 serving a dual role as a core molecule that amplifies the immune-suppressive function of TAMs while dampening the anti-tumor immune response (Huang et al., 2020). In addition, CSC can recruit M-MDSC via CD74 via macrophage migration inhibitory factor (MIF) (Wang et al., 2016; Alban et al., 2020).

Regulatory T cells (Treg cells), as a major subset of suppressor T cells, play a central role in tumour immunology. Self-renewing CSC express high levels of indoleamine 2,3-dioxygenase 1 (IDO1) and TGFβ from a variety of cancer cell lines, both of which are key inducers of Treg cell recruitment and generation (Wainwright et al., 2012; Stapelberg et al., 2014; Nakano et al., 2019; Shidal et al., 2019; Ozawa et al., 2020). Through the production of these mediators, CSC are able to stimulate Treg cells, further enhancing the expression of CSC markers and self-renewal. CSC actively recruit Treg cells to the tumour microenvironment through the secretion of chemokines, such as CCL1, CCL2, and CCL5 (Chang et al., 2016; Xu et al., 2017; Chen et al., 2018; You et al., 2018; Su et al., 2019), a process that has been validated in a mouse glioblastoma model, in which CCL2 relies on the expression of CCR4 to mediate the Treg cell trafficking (Chang et al., 2016). In human ovarian cancer cell lines, CD133+ cells with high CCL5 expression induced Treg cell migration and promoted IL-10 production in a CCR5-dependent manner (You et al., 2018). In contrast, CCL1 in SOX2+ mouse breast cancer cell lines induced Treg cell migration, suggesting that multiple chemokines are involved in the recruitment of Treg cells to the tumour microenvironment.

The interaction of Tregs with CSCs further complicates the tumour immune microenvironment, e.g., in malignant gliomas, CSCs enhance the latter’s aggregation and immunosuppressive efficacy by exploiting the interaction of B7-H1 with Tregs to co-create an immunosuppressive atmosphere. Some Tregs also directly eliminated NK cells and CTLs, exacerbating tumour immune escape and driving tumour progression (Xu et al., 2017).

These findings highlight the complex network of interactions between TAMs and Tregs in regulating the tumour microenvironment, promoting CSC maintenance and immune escape, and provide important targets for the development of targeted cancer therapeutic strategies.

#### 5.2.2 Secretion of immunosuppressive factors

Cancer stem cells (CSC) play a dual role in the immune system by recruiting immune cells for their own benefit while also suppressing the anticancer activity of T cells and natural killer (NK) cells through the secretion of immunosuppressive factors. This not only impairs the anti-tumor function of macrophages but also leads to their transformation into an immunosuppressive phenotype, serving as a key mechanism for CSC immune evasion (Lei and Lee, 2021).

Studies have shown that CSCs of different origins achieve immune escape through the expression of specific molecules, and that CSCs upregulate CD80 expression in a TGFβ-dependent manner, thereby interfering with the normal function of T cells (Miao et al., 2019). Lymphoma stem cells, pancreatic cancer stem cells and lung cancer stem cells highly express CD47, a protein that binds to SIRPα on the surface of macrophages and sends a “do not engulf me” signal, which effectively inhibits macrophage phagocytosis and helps CSCs to evade immune clearance (Jia et al., 2021). In addition, CSC in prostate, breast, colon and brain tumours tend to highly express CD200, which belongs to the immunoglobulin superfamily, and by interacting with CD200R, it can inhibit the activity of myeloid cells, such as monocytes and macrophages, and promote the shift of immune response from pro-inflammatory Th1 to anti-inflammatory Th2, further reducing the strength of the body’s immune response (Liao et al., 2013).

In studies of glioblastoma, cancer stem cells (CSC) have been observed to express high levels of the biological clock-regulating protein CLOCK. This protein forms a complex with BMAL1, promoting stem cell self-renewal by increasing the expression of OLFML3. Additionally, this interaction indirectly enhances the ability of tumors to evade the immune system by attracting immune-suppressing microglial cells to the tumor microenvironment.

Therefore, CSC plays a crucial role in finely modulating the immune microenvironment and inhibiting anti-tumor immune responses through various mechanisms, such as the expression of CD47, CD200, the CLOCK/BMAL1/OLFML3 pathway, and IL1R2. These discoveries not only advance our understanding of how CSC evades the immune system but also lay the groundwork for the development of novel immunotherapeutic strategies and potential targets for intervention.

### 5.3 Evasion of immune system clearance by high expression of immune checkpoint molecules etc.

Cancer stem cells (CSC) can upregulate immune checkpoint molecules like PD-L1, CTLA-4, B7-H3, and B7-H4, enabling them to evade immune surveillance more effectively (Mortezaee and Majidpoor, 2023). Among these, PD-L1 and CTLA-4 are the most well-known immune checkpoint molecules (Zhang et al., 2021).

#### 5.3.1 High expression of PD-L1 inhibits T cell activity

The effect of CSCs on T cell activity is a more complex and intensively studied process that encompasses multiple dimensions of direct and indirect exposure. Compared to normal tumour cells, CSCs impair cytotoxic T cell activity by selectively enriching for inhibitory checkpoint ligands. Although all co-inhibitory receptors expressed by CSCs have not been fully explored, it has been noted that phenotypic markers defining CSCs are often accompanied by higher levels of inhibitory checkpoint receptors. For example, PD-L1 expression is increased in CD44^+^ breast cancer stem cells (Wu et al., 2017; Hsu et al., 2018), CD133+ colorectal cancer stem cells (Zhi et al., 2015; Wu et al., 2017), and CD44^+^ head and neck squamous cell carcinoma (HNSCC) stem cells; VTCN1 expression is also elevated in CD133+ glioblastoma stem cells.

PD-1 and its ligand PD-L1 are crucial immune checkpoint molecules that play a central role in allowing tumor cells to evade detection by the immune system. When PD-L1 on tumor cells binds to PD-1 on T cells, it hinders T cell proliferation and promotes cell death, ultimately suppressing the body’s ability to mount an effective anti-tumor immune response (Zhou et al., 2023). Studies have demonstrated that the interaction between PD-1 and PD-L1 leads to a metabolic shift in T cells, characterized by reduced glycolysis and amino acid metabolism, and increased fatty acid oxidation (Kumagai et al., 2022). This altered metabolic state is closely linked to T cell dysfunction, impacting the activity of CD8^+^ cytotoxic T cells and CD4^+^ helper T cells while also promoting the growth of regulatory T cells (Tregs) and T cell depletion.

Blocking the PD-1/PD-L1 pathway can restore T cell function and viability. By using antibodies known as immune checkpoint inhibitors against PD-1 or PD-L1, it is possible to counteract the inhibitory effects of PD-1 on T-cell receptor (TCR) signaling. This reactivation of depleted CD8^+^ effector T cells enhances their ability to mount an effective anti-tumor immune response (Ai et al., 2020). Furthermore, this intervention can partially reverse the unfavorable metabolic changes observed in T cells, such as restoring glycolysis and amino acid metabolism while reducing fatty acid oxidation. This restoration promotes normal T cell differentiation and memory formation, ultimately boosting the immune system’s ability to fight cancer.

In recent years, there have been a limited number of studies focusing on the impact of the PD-1/PD-L1 pathway in tumor stem cells. Schatton et al. (2010) introduced the concept that tumor stem cells have the ability to suppress T cell activity. They observed an upregulation of PD-L1 expression in stem cells of head and neck malignant tumors. The presence of PD-L1 in tumor cells and antigen-presenting cells within the tumor microenvironment was shown to hinder the activation of tumor-specific T cells and impede the immune response against tumors through the PD-1/PD-L1 signaling pathway. This implies that tumor stem cells might impede the immune response against tumors mediated by T cells through the PD-1/PD-L1 pathway.

Yan et al. utilized the gastric cancer SGC-7901 cell line derived from pre-chemotherapy gastric cancer tissues, which were cultured under serum-free conditions to induce the formation of stem cell spheres. These stem cells exhibited a higher resistance to different concentrations of 5-FU compared to normal gastric cancer cells. The researchers observed PD-L1 expression in these tumor cells, with a higher expression of the proliferation-associated antigen Ki67 in PD-L1-positive tumor stem cells. The study also found an upregulation of Ki67 expression in the group stimulated with PD-1 Ig, leading to faster tumor growth in mice injected with these cells. This indicates that PD-L1 acts as a stimulatory signal for the proliferation of tumor stem cells, and the presence of the PD-1/PD-L1 signaling pathway enhances the proliferative capacity of tumor stem cell spheroids. The combined use of anti-PD-1 and anti-PD-L1 antibodies to block the PD-1/PD-L1 pathway specifically can hinder tumor stem cell proliferation and decrease tumor recurrence rates, showing promise for gastric cancer treatment. Seow et al. also confirmed in their study of immunotherapy for colorectal cancer that the use of anti-PD-1/PD-L1 antibody, which closes the blocking of the PD-1/PD-L1 pathway, attenuates the tumor feasibility of tumorigenic effects of stem cells.

#### 5.3.2 Inhibition of T cell activity by high expression of CTLA-4

CTLA-4, also known as cytotoxic T-lymphocyte-associated antigen four or CD152, is a transmembrane protein primarily found in activated T cells and natural killer (NK) cells (Gardner et al., 2014). It acts as a homodimeric receptor on T cells, interacting with B7-1 (CD80) and B7-2 (CD86) on antigen-presenting cells (APCs) (Esensten et al., 2016; Tekguc et al., 2021). By competing with CD28 for binding to the B7 molecule, CTLA-4, with its higher affinity for the ligand, inhibits T-cell activation and transmits inhibitory signals to the T cell, ultimately down-regulating T cell responses (Wang et al., 2015).

This immune checkpoint molecule plays a crucial role in regulating T cell-mediated immune responses, potentially leading to T cell exhaustion and dysfunction, thus dampening the body’s immune response to tumor cells. For instance, melanoma stem cells use CTLA-4 to induce tumor cell proliferation and inhibit apoptosis, while cutaneous squamous cell carcinoma stem cells activate CTLA-4 expression through CD80, hindering the activity of cytotoxic T-lymphocytes (CTLs) and aiding tumor cells in evading immune surveillance.

Blocking CTLA-4, such as with inhibitors like Ipilimumab, can reverse the inhibitory effect on T cells, reactivating their immune response against tumors and producing an anti-tumor effect (Buchbinder and Desai, 2016). Ipilimumab, an FDA-approved inhibitor of CTLA-4, disrupts the B7-CD28 interaction by blocking CTLA-4, thereby boosting T cell immune responses against tumor cells. This immune checkpoint inhibitor offers a novel approach to tumor treatment by enhancing the body’s own anti-tumor immunity.

#### 5.3.3 High expression of B7-H3

The immune checkpoint molecule B7-H3, also known as CD276, belongs to the B7-CD28 family and is overexpressed in various malignant tumors, correlating with prognosis. Its expression directly impacts immune cells in the tumor microenvironment, playing a crucial role in tumor development. B7-H3 regulates epithelial mesenchymal transition (EMT) and tumor stem cell-like (CSC) properties by modulating the expression of specific proteins. Inhibition of B7-H3 reduces tumor-initiating cells and suppresses glioma cell invasion and sphere formation. Tumor stem cells, resistant to chemotherapy, exhibit CSC properties induced by B7-H3. This molecule also contributes to chemoresistance in breast and colorectal cancer cells by activating the JAK2/STAT3 pathway (Zhang et al., 2015). Overall, B7-H3 is a key immune checkpoint molecule involved in tumor aggressiveness and chemoresistance, highlighting its potential as a therapeutic target in cancer treatment. The interaction between CSCs and immune cells, mediated by immune checkpoint molecules like PD-L1 and CTLA-4, underscores the importance of targeting these molecules in tumor immunotherapy.

### 5.4 The role of activation of immune tolerance signaling pathways in immune escape of CSCs

Cancer stem cells’ own oncogenic signaling pathways are also significant in immune evasion. These specific pathways not only stimulate tumor growth and survival, but also hinder the normal function of immune cells, contributing to immune escape through a complex mechanism.

#### 5.4.1 Wnt/β-catenin signaling pathway

This pathway is aberrantly activated in several types of cancers and not only drives tumour development, but is also associated with the phenomenon of immune rejection. In glioblastoma and melanoma, activation of β-catenin signalling was observed to correlate with the absence of T-cell infiltration in the tumour microenvironment. In studies in a mouse model of melanoma, β-catenin signalling within the tumour prevented the recruitment of dendritic cells (DCs) by inhibiting the expression of the chemokine CCL4, which in turn impeded the process of T-cell activation against tumour antigens (Spranger et al., 2015; Yang et al., 2020).

In addition, a significant increase in PD-L1 expression was observed in breast mesenchymal-like cancer cells, especially breast cancer stem cells (CSCs), when breast cancer cells underwent epithelial mesenchymal transition (EMT) through the regulation of the EMT/β-catenin/STT1/PD-L3 signalling pathway. It was shown that the ER-associated N-glycosyltransferases STT3A and STT3B play a key role in the stability of PD-L1 by regulating its glycosylation, which in turn antagonises β-TrCP-dependent proteasomal degradation and ensures stable PD-L1 expression (Hsu et al., 2018). Etoposide inhibits the EMT/β-catenin/STT1/PD-L2 signalling axis through TOP3B-dependent β-catenin degradation, leading to downregulation of PD-L1 in breast cancer stem cells (CSCs) and non-stem cells (non-CSCs). This process enhances the sensitivity of cancer cells against Tim-3 treatment.

Overall, evidence suggests that the WNT/β-catenin signalling pathway is closely linked to immune escape mechanisms, promoting tumour immune escape by regulating the “do not find me” signal PD-L1 and other molecules involved in immune control. This finding provides new insights into the mechanism of tumour immune escape and may open the way for the development of cancer immunotherapy strategies targeting the WNT/β-catenin signalling pathway.

#### 5.4.2 NOTCH signaling pathway

The Notch pathway plays a crucial role in regulating gene expression and determining the fate of cancer stem cells (CSCs) by mediating interactions between the Notch receptor and its ligand. Activation of the Notch pathway, through γ-secretase cleavage of the receptor, leads to the release of intracellular fragments that influence key factors like NF-κB, impacting the stemness of CSCs. Inhibition of γ-secretase disrupts CSC properties and hinders tumor development. Aberrant Notch signaling activation is linked to the malignant transformation of tumors and the promotion of self-renewal in breast CSCs via factors like IL-6. While miR-1275 activates the Notch pathway to sustain lung cancer CSC stemness, compounds such as quercetin, cucurbitacins B and I inhibit this pathway, reducing the number of colon cancer CSCs. Abnormal activation of the NOTCH signaling pathway in various cancer types is associated with distinct immunosuppressive characteristics. For instance, in colorectal cancer, NOTCH2 activation is linked to tumor-associated macrophages (TAMs) favoring M2-type polarization, known for immunosuppression and a pro-tumor microenvironment. Conversely, in pancreatic cancer, NOTCH2 amplification is correlated with reduced activity of cytotoxic T lymphocytes (CTLs).

#### 5.4.3 YAP1 signaling pathway

YAP1, as a key downstream effector of the Hippo signaling pathway, has been linked to immune evasion in various cancers. In ovarian cancer, elevated YAP1 expression is correlated with reduced CTL infiltration, indicating a potential role of YAP1 in hindering immune surveillance by modulating the tumor microenvironment. Conversely, in prostate cancer, heightened YAP1 activity is linked to enhanced recruitment of myeloid-derived suppressor cells (MDSCs), crucial immunosuppressive cells that dampen both adaptive and innate immune reactions.

Studies have shown that cancer stem cell-like cells (CSLCs) exhibit enhanced immune escape in lung adenocarcinoma (LUAD), which is closely associated with the expression of signal-regulated protein γ (SIRPγ). SIRPγ promotes the dephosphorylation of MST1 by mediating the interaction between MST1 and PP2A, thereby activating the Hippo/YAP signalling pathway. The activation of the Hippo/YAP signalling pathway contributes to the activation of the MST1/YAP signaling pathway. The activation of YAP signalling pathway prompted CSLC to release cytokines, which further upregulated the expression of CD47, thus effectively inhibiting the phagocytosis of tumour cells (Wang et al., 2016; Sarkar et al., 2017).

## 6 Summary and outlook

Since the initial discovery of Cancer Stem Cells (CSCs) in hematological tumors approximately 30 years ago, there has been significant advancement in their study. CSCs are found in various types of tumors and have emerged as potential therapeutic targets for numerous malignancies, offering novel avenues for cancer treatment. CSCs undergo evolution during tumor progression, giving rise to distinct groups with varying capabilities, such as pre-CSCs, pri-CSCs, mig-CSCs, cr-CSCs, and rr-CSCs. These groups play crucial roles in tumorigenesis, progression, metastasis, and resistance to radiotherapy and chemotherapy, with potential for interconversion. Understanding the origin and classification of CSCs serves as a theoretical foundation for targeted therapeutic strategies. Research on CSC surface markers enables more precise screening and isolation of CSCs, establishment of CSC models, and exploration of their molecular mechanisms. Additionally, CSCs exhibit unique microenvironmental characteristics and metabolic reprogramming that drive tumorigenesis, progression, metastasis, and recurrence.

Targeting the metabolic vulnerabilities of CSCs and modulating their microenvironment hold promise for innovative cancer therapies. As the instigators of tumorigenesis, CSCs can suppress the growth and function of immune cells, alter their phenotype, and hinder their anti-tumor activities through various pathways. CSCs can orchestrate an immunosuppressive microenvironment, evade immune surveillance, and promote tumor growth and recurrence. By targeting the immune evasion mechanisms of CSCs, enhancing immune response, disrupting the immunosuppressive microenvironment, inhibiting overexpressed immune checkpoints, and blocking CSC-specific oncogenic signaling pathways, it may be possible to eradicate CSCs, impede tumor progression, and prevent recurrence. Therefore, in-depth exploration of the biological and immunological properties of CSCs and isolation of specific antigens are of great significance to the study of CSCs and the search for effective therapeutic targets.

Nevertheless, the study of CSCs is still in its infancy, and many problems need to be solved. The isolation of CSCs remains a key challenge in research, primarily relying on surface markers that are specific to CSCs, yet only a limited number of such markers have been identified and validated. Further investigations are necessary to uncover distinct surface markers unique to CSCs compared to normal stem cells. Additionally, the heterogeneity of tumors, largely driven by CSCs, results from intricate interactions within the tumor microenvironment, leading to diverse metabolic changes and heterogeneity in the tumor microenvironment. Environmental stresses can prompt adaptive alterations in tumor cells, while abnormal activation or inhibition of cell signaling pathways further contributes to CSC heterogeneity. However, a comprehensive understanding of the internal spatial structure of tumors is currently lacking, hindered by technical constraints in monitoring dynamic internal changes and a scarcity of suitable animal models for in-depth exploration of tumor heterogeneity and evolution. Despite these challenges, as research in the field of CSCs progresses, it is anticipated that advancements will be made in overcoming these obstacles and achieving breakthroughs in tumor-targeted therapies.
